# Thermal-tolerant potential of ordinary *Chlorella pyrenoidosa* and the promotion of cell harvesting by heterotrophic cultivation at high temperature

**DOI:** 10.3389/fbioe.2022.1072942

**Published:** 2022-12-01

**Authors:** Yu-Ren Dai, Die Wang, Yu-Rong Zhu, Kun-Xiao Yang, Ning Jiao, Zhong-Liang Sun, Shi-Kai Wang

**Affiliations:** ^1^ Joint International Research Laboratory of Agriculture and Agri-Product Safety, Yangzhou University, Yangzhou, China; ^2^ College of Life Sciences, Yantai University, Yantai, China

**Keywords:** *Chlorella pyrenoidosa*, temperature, heterotrophic culture, cell harvesting, cell size, cell surface charge

## Abstract

During the heterotrophic cultivation of microalgae, a cooled process against temperature rise caused by the metabolism of exogenous organic carbon sources greatly increases cultivation cost. Furthermore, microalgae harvesting is also a cost-consuming process. Cell harvesting efficiency is closely related to the characteristics of the algal cells. It may be possible to change cell characteristics through controlling culture conditions to make harvesting easier. In this study, the mesophilic *Chlorella pyrenoidosa* was found to be a thermal-tolerant species in the heterotrophic mode. The cells could maintain their maximal specific growth rate at 40°C and reached 1.45 day^−1^, which is equivalent to that of cultures at 35°C but significantly higher than those cultured at lower temperatures. Interestingly, the cells cultured at 40°C were much easier to be harvested than those at lower temperatures. The harvesting efficiency of the cells cultured at 40°C reached 96.83% after sedimentation for 240 min, while the cells cultured at lower temperatures were reluctant to settle. Likely, the same circumstance occurred when cells were harvested by centrifugation or flocculation. The promotion of cell harvesting for cells cultured at high temperatures was mainly attributed to increased cell size and decreased cell surface charge. To the best of our knowledge, this is the first report that cells cultured at high temperatures can promote microalgae harvesting. This study explores a new approach to simplify the cultivation and harvesting of microalgae, which effectively reduces the microalgae production cost.

## 1 Introduction

Microalgae have been recognized as promising alternative sources for the accumulation of high-value compounds, such as lipids, proteins, carbohydrates, and pigments, which can be used for the production of biofuels, single-cell protein (SCP), animal feed, and food supplements (Janssen et al., 2022; Show, 2022). However, the high production cost and low production capacity greatly inhibit its commercial application. It is necessary to develop an efficient and economic approach for the abundant production of microalgal biomass. When compared to photoautotrophic cultivation, heterotrophic cultivation eliminates the light requirement; simplifies the design, operation, and scale-up of a bioreactor; and greatly boosts the biomass productivity (Wang et al., 2018). Under fed-batch heterotrophic fermentation of *Chlorella sorokiniana* GT-1, the biomass concentration reached as high as 271 g/L in 7.5 L bench-scale fermenters and 247 g/L in 1,000 L pilot-scale fermenters (Jin et al., 2021). Heterotrophic cultivation shows a great potential for economic and abundant production of microalgae.

During heterotrophic cultivation of microalgae, the metabolism of exogenous organic carbon sources generates a lot of heat, leading to a rise in the temperature of the culture. To maintain the optimal growth temperature, it is necessary to control temperature through cooling, but this process is usually expensive (Barten et al., 2022). It has been estimated that a cost reduction by 26.2% and 28.4% could be achieved if the algal cells could maintain their maximal productivity rate under high temperatures from 30 to 40 and 45°C, respectively (Ruiz et al., 2016). Temperature is one of the most important environmental parameters, impacting algal cellular physiology by changing the rate of chemical reactions and the stability of cellular components (Wang et al., 2016). Most industrial microalgal species have an optimal growth temperature ranging from 15 to 25°C, and the maximal growth temperature is usually between 25 and 35°C (Abdelaziz et al., 2014; Barten et al., 2022). Within a certain temperature range, the cell growth rate is enhanced by increasing the temperature, following the logic of the Arrhenius law. However, when exceeding the optimal growth temperature, the structure of some proteins, especially those in the electron transport chain, would be changed, leading to a rapid drop in cell growth rate concomitant with increased mortality (Serra-Maia et al., 2016). It would be helpful to reduce culture costs by screening algal species that are naturally resistant to stressful temperatures.

Harvesting algal cells from the culture broth is always considered a major obstruction for algal biomass production, which is the main limiting factor for its commercial application (Weschler et al., 2014). Due to the dilute nature of the algal broth, small cell size, and electronegative surface charge of most algal cells, the typically used harvesting methods, such as centrifugation, sedimentation, flocculation, filtration, flotation, or a combination of these methods, usually face various problems, such as high energy consumption, time consumption, and high cost (Wang et al., 2015). The cell harvesting efficiency is closely related to the characteristics of the algal cells, such as its morphology, motility, surface charge, cell mass density, and extracellular organic matter composition and concentration (Henderson et al., 2008). Cell characteristics are greatly influenced by environmental factors. It may be possible to change cell characteristics by controlling the culture conditions to make harvesting easier. However, no related studies have been reported.

In this study, mesophilic *Chlorella pyrenoidosa* was detected to have gained excellent heat resistance in heterotrophic cultures. More importantly, the cells cultured at high temperatures showed higher settlement efficiency. To explore the feasibility of promoting microalgae harvesting by high-temperature cultures, *C. pyrenoidosa* was heterotrophically cultured at different temperatures. The cell growth and bioproducts accumulation of the cultures at different temperatures were analyzed. Afterward, the algal cells cultured at different temperatures were harvested at the stationary phase by the commonly used separation methods, which included gravity sedimentation, centrifugation, and flocculation, to compare their harvesting performance. The main factors affecting cell harvesting were further clarified. This study has indicated that heterotrophic high-density fermentation at a high temperature might be an effective approach for efficient cultivation and simple harvesting of microalgae.

## 2 Materials and methods

### 2.1 Microorganism strains and inoculum culture

*Chlorella pyrenoidosa* FACHB-9 was purchased from the Freshwater Algae Culture Collection at the Institute of Hydrobiology, Chinese Academy of Sciences. The inoculum culture used liquid BG-11 medium with 5 g/L glucose and was incubated at 25°C in an orbital shaker at 150 rpm under dark conditions. After being cultured for 4 days, it was inoculated in the following experiments.

### 2.2 Microalgae culture

The cultures were first conducted in a 250 ml Erlenmeyer flask with a 100 ml working volume using the modified Endo medium (Endo et al., 1974). The initial concentration of glucose was chosen to be 20 g/L on the basis of the preexperimental results. The precultured cells were inoculated into an Erlenmeyer flask with an inoculum size of the OD_680nm_ at 0.1. To investigate the effects of temperature on the growth of *C. pyrenoidosa*, the cultures were incubated at 25, 30, 35, 40, 42, and 45°C, respectively. All the cultures were conducted on an orbital shaker at a speed of 150 rpm in dark conditions.

### 2.3 Cell harvesting

When the growth reached the stationary phase, the culture broth was directly tested for cell harvesting without any treatment. The harvesting efficiency of the cells cultured at different temperatures was tested and compared using three commonly used harvesting methods, which include gravity sedimentation, flocculation, and centrifugation. Gravity sedimentation was carried out in a 100 ml beaker with 50 ml culture broth. Then, 200 μl of the sample was taken from the center of the broth to measure the optical density at 680 nm at different time intervals. Chitosan was used as the flocculant in the flocculation test. Chitosan (Sinopharm, China) was first dissolved in 0.1% acetic acid. Then, it was diluted to make the final chitosan concentration of 5 g/L using deionized water. The flocculation process was operated according to the method modified from Rashid et al. (2013). Briefly, a specific amount of chitosan was added to and mixed with the culture broth at 50 rpm for 2 min. Then, the algal cells were allowed to settle down for 3 min. Two hundred microliters of the sample was taken from the center of the broth to measure the optical density at 680 nm. A centrifuge (Cence H1850, China) was used for the centrifugation test. The centrifugation time was set to 2 min, and the centrifugation speed was set from 1,000 to 4,500 rpm. The relative centrifugal force (RCF, *g*) was calculated as shown in Eq. 1:

$$RCF=1.118\times 10^{-5}\times n^{2}\times r,$$ (1)

where n and r are the centrifugation speed (rpm) and centrifugal radius (cm), respectively.

After centrifugation, the optical density at 680 nm of the supernatant was measured. The harvesting efficiency (R, %) in the aforementioned experiments was calculated as follows:

$$R\left(\%\right)=\left(1-\frac{{OD}_{t}}{{OD}_{0}}\right)\times 100,$$ (2)

where OD_0_ and OD_t_ are the optical densities at time zero and time t, respectively.

All harvesting experiments were carried out at room temperature.

### 2.4 Analytic methods

Algal cells were first harvested by centrifugation at 8,000 rpm for 10 min. After being washed twice with distilled water, they were lyophilized using a lyophilizer (FD-1A-50, BiLon, China). The total biomass concentration in each culture was measured by the gravimetric method. The glucose concentration was measured by the 3,5-dinitrosalicylic acid (DNS) method. The protein content was measured according to the method reported by Tian et al. (2020). Lipid extraction and quantification were performed according to the methods reported by Wang et al. (2020). For the measurement of starch content, the lyophilized cells were first ground for 10 min using a mortar and pestle and then measured using a starch detection kit (BC0700, Solarbio, China) according to the manufacturer’s instructions. The morphology of *C. pyrenoidosa* was observed using a microscope (Mshot ML31, China). For the analysis of cell size, more than 300 cells were randomly chosen and their diameters were measured using a microscope (MSHOT ML31, China). The size distribution of cells cultured at different temperatures was analyzed using Origin 9 (OriginLab, United States). The zeta potential of the cultures on the 5th day was measured using a Malvern Zetasizer Nano ZS90.

### 2.5 Statistical analysis

Data are presented as mean ± standard error of the mean based on three parallel experiments. The statistical significances were analyzed by one-way analysis of variance (ANOVA) (*p* < 0.05) using Origin 9 (OriginLab, United States).

## 3 Results and discussion

### 3.1 Effect of temperature on growth and glucose consumption of *Chlorella pyrenoidosa*

**FIGURE 1:**
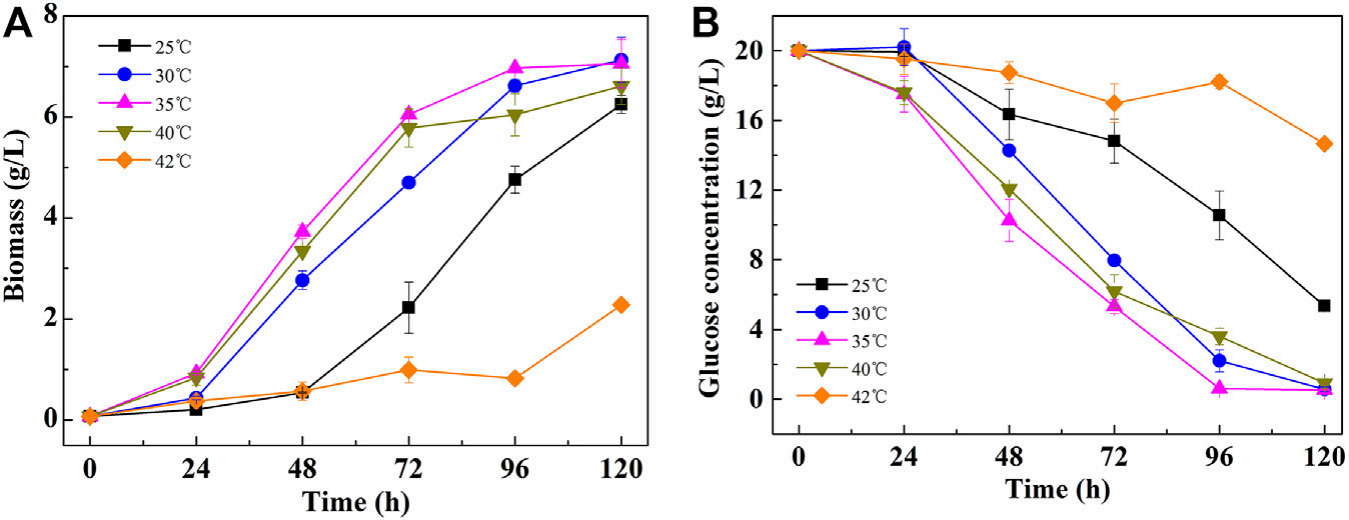
Growth curves **(A)** and glucose consumption **(B)** of *C. pyrenoidosa* cultured at different temperatures.

As shown in Figure 1A, *C. pyrenoidosa* exhibited excellent heat resistance. With temperatures increasing from 25 to 35°C, the maximal specific growth rate increased from 0.89 to 1.47 day^−1^. Interestingly, the cells also grew well at 40°C, in which the specific growth rate reached 1.45 day^−1^, which is equivalent to the cells grown at 35°C (Table 1). However, when the temperature was further increased to 42°C, the cell growth became significantly inhibited and the culture broth turned yellow. At 45°C, the cell growth completely ceased and the cells gradually died. The biomass production in the cultures at 40°C reached 6.61 g/L, which was only slightly lower than that at the highest temperature, 7.13 g/L at 30°C.

**TABLE 1:** Parameters of growth kinetics and cell composition of cells cultured at different temperatures.

Temperature (°C)	Final biomass (g/L)	Maximal specific growth rate (day^−1^)	Protein content (%)	Lipid content (%)	Carbohydrate content (%)
25	6.25 ± 0.18 b	0.89 ± 0.01 c	38.80 ± 2.69 a	21.04 ± 0.99 c	39.85 ± 0.07 b
30	7.13 ± 0.45 a	1.12 ± 0.01 b	32.75 ± 0.49 b	23.71 ± 0.57 a	39.05 ± 0.35 b
35	7.06 ± 0.48 a	1.47 ± 0.01 a	39.65 ± 3.47 a	22.34 ± 0.14 b	32.65 ± 0.78 d
40	6.61 ± 0.36 b	1.45 ± 0.02 a	38.55 ± 1.29 a	20.13 ± 0.42 c	34.65 ± 1.34 c
42	2.28 ± 0.07 c	0.68 ± 0.01 d	35.95 ± 0.34 b	16.42 ± 0.28 d	44.25 ± 0.94 a

Values in a column with different letters are significantly different according to one-way analysis of variance (ANOVA) (*p* < 0.05).

Similar to the cell growth trend, the glucose consumption rate was also promoted with the temperature increasing from 25 to 35°C. The glucose was exhausted after 108 and 96 h in cultures at 30 and 35°C, respectively. However, even after 120 h of incubation, the remaining glucose was still as high as 5.35 g/L in cultures at 25°C (Figure 1B). At 40°C, the glucose consumption rate at an early stage followed the same trend as the ones at 35°C, and then it slowed down with decreasing cell growth rate. As the cell growth was strongly inhibited at 42°C, only a small amount of glucose was utilized under this condition.

*Chlorella pyrenoidosa* is generally regarded as a mesophilic species when cultured in an autotrophic mode. Currently, there is no report on the tolerance of wild-type *Chlorella* to high temperatures. It usually cannot grow at temperatures above 35°C (Sachdeva et al., 2016). The reported highest temperature for *C. pyrenoidosa* has been 30°C in either autotrophic or heterotrophic cultures (Fan et al., 2014; Dong et al., 2020). Zhang L et al. (2020) reported that the addition of exogenous salicylic acid distinctly alleviated the inhibition of heat stress on *Nannochloropsis oceanica*. In addition, adaptive laboratory evolution (ALE) has been proven to be effective in improving the thermal tolerance of many algal species. For example, Huertas et al. (2011) found that *Scenedesmus intermedius* could grow rapidly at 40°C after gradually adapting to this temperature for 135 generations, while its ancestral strain could not survive at this condition. Barten et al. (2022) increased the maximum mid-day growth temperature of a thermos-tolerant microalgae *Picochlorum* sp. (BPE23) from 47.5 up to 49°C by using ALE. However, the adaptation process usually requires many generations of evolution or long-time processing (Huertas et al., 2011; Barten et al., 2022). Random mutagenesis is more efficient than ALE for screening thermo-tolerant species. Two thermo-tolerant mutants of *C. pyrenoidosa* NCIM 2738 were obtained by using ethyl methane sulfonate mutagenesis treatment. They were capable of surviving at temperatures up to 47°C and showed improved lipid and biomass yields, while the wild type could not survive at 35°C (Sachdeva et al., 2016). Although this method is efficient and simple, the workload is heavy. Algal cells can be acclimated to rebalance cellular homeostasis to counteract the adverse effects of increased temperature. This mainly includes the rebalancing of cell composition and elevation of cell membrane fluidity (Barten et al., 2021). When compared to autotrophic cultures, algal cells were more tolerant to high temperatures under the heterotrophic mode. Li et al. (2013) found that the optimal growth temperature for the heterotrophic culture of wild *C. sorokiniana* (UTEX 1602) was 37°C; in addition, the specific growth rates at 40 and 42°C reached 1.26 and 1.35 day^−1^, respectively, which is significantly higher than its counterpart at 21°C. This result indicated that the microalga *C. sorokiniana* could tolerate temperatures as high as 42°C, which is similar to this study. In this study, the naturally occurring wild type of *C. pyrenoidosa* showed excellent heat resistance at temperatures up to 40°C under heterotrophic cultures. *Chlorella pyrenoidosa* (FACHB-9) has been extensively studied in many aspects, but this is the first report involving its heat resistance; it might be possible that its ability of heat resistance in the heterotrophic culture mode might have been neglected.

### 3.2 Effect of temperature on cell composition of *Chlorella pyrenoidosa*

The accumulation of proteins, lipids, and carbohydrates in cultures at different temperatures is summarized in Table 1. The protein content of cells cultured at 40°C reached 38.55%, which is comparable to the ones cultured at 25 and 35°C, but the protein content from the cultures at 30 and 42°C is significantly lower than that at other conditions. The highest lipid content of 23.71% was achieved at 30°C. Further temperature increase after 30°C led to a continuous decrease in lipid content. It reached 20.13% in cultures at 40°C. The lowest carbohydrate content was obtained at 35°C. It was enhanced either by increasing or decreasing the culture temperature. The highest carbohydrate content of 44.25% was obtained in cells cultured at 42°C. Combined with the biomass productivity, the protein productivity of cultures at 40°C reached 2.55 g/L/day, which is significantly higher than that of cultures at other temperatures except for cultures at 35°C. Meanwhile, the lipid productivity in cultures at 40°C was 1.33 g/L/day, which is lower than that of cultures at 30 and 35°C.

It has been widely reported that the biochemical composition is greatly influenced by culture temperature. For the accumulation of a specific product, temperature sometimes has a greater impact than the other factors, such as light intensity and inoculum size (Zheng et al., 2022). A decreasing trend in protein accumulation was found in *Spirulina maxima* and *Spirulina platensis* when increasing the temperature from 20 to 40°C (De Oliveira et al., 1999). Zhang Z et al. (2020) also found that low temperature was beneficial for the formation of protein during the mixotrophic cultivation of *Chlorella vulgaris*. However, Sayegh and Montagnes (2011) pointed out that there are no consistent trends in the change of protein with temperature, although significant temperature isolated interactions existed. Similar circumstances have also been detected in lipid formation. Chaisutyakorn et al. (2018) found that the lipid content of three tested marine microalgae was decreased with increasing temperatures from 25 to 40°C. Sonmez et al. (2016) reported that the highest lipid content was obtained at 25°C in the cultures of *Micractinium* sp. and *Scenedesmus* sp. It was greatly decreased when the temperature was increased to 30°C. These results are consistent with this study, indicating that the high temperature was unfavorable for lipid accumulation. However, in cultures of *C. vulgaris* and *Scenedesmus* sp., the temperatures below the optimal growth temperature were favorable for lipid accumulation (Converti et al., 2009; Li et al., 2011). De Oliveira et al. (1999) found that the lipid content in *Spirulina maxima* and *Spirulina platensis* was increased with increasing temperatures from 20 to 40°C. The change in temperature caused an imbalance between energy supply and consumption, and then the inherent biochemical or physiological functions changed accordingly (Ras et al., 2013). The variations in biochemical composition at different temperatures was a cellular adaptive process (Barten et al., 2021). Collectively, biochemical accumulation is effected by temperature in diverse ways, and there are no consistent trends across species. The accumulation rules of target products should be considered when choosing the culture temperature.

### 3.3 Effect of culture temperature on cell harvesting

**FIGURE 2:**
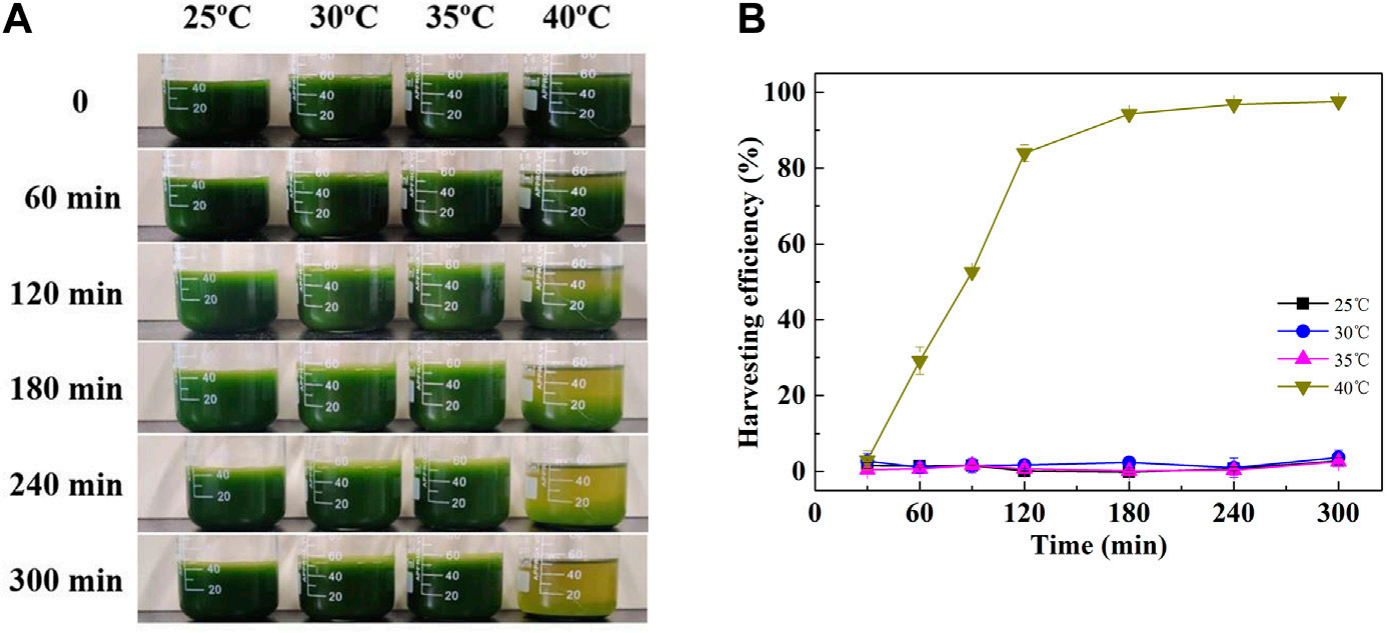
Gravity sedimentation process **(A)** and sedimentation efficiency **(B)** of *C. pyrenoidosa* cultured at different temperatures.

By the end of the culture, it was occasionally identified that the cells cultured at high temperatures were prone to sedimentation. As shown in Figure 2, the harvesting efficiency of cells cultured at 40°C was gradually increased with time. It reached 94.27% and 96.83% after sedimentation for 180 and 240 min, respectively. However, cells cultured at lower temperatures (25, 30, and 35°C) were reluctant to settle (Figure 2A). The highest harvesting efficiency was no more than 4% at these conditions even for more than 300 min of sedimentation (Figure 2B). Although cell recovery by gravity sedimentation is an additive-free and low-cost harvesting method, it is only suitable for relatively large microalgae (Li et al., 2020). Microalgae with small size, for example, *Chlorella* sp., were usually hard to be harvested only by gravity sedimentation (Hong et al., 2022). To enhance the settling velocity, it is common to apply a coagulation or flocculation step prior to gravity sedimentation. Coagulation or flocculation can be induced by pH adjustment or the addition of flocculants (Hapońska et al., 2018; Hong et al., 2022). In addition, microalgae with autoflocculation ability could achieve a higher settling velocity and exhibit a better potential, as this process dispensed with the addition of flocculants (Li et al., 2021).

**FIGURE 3:**
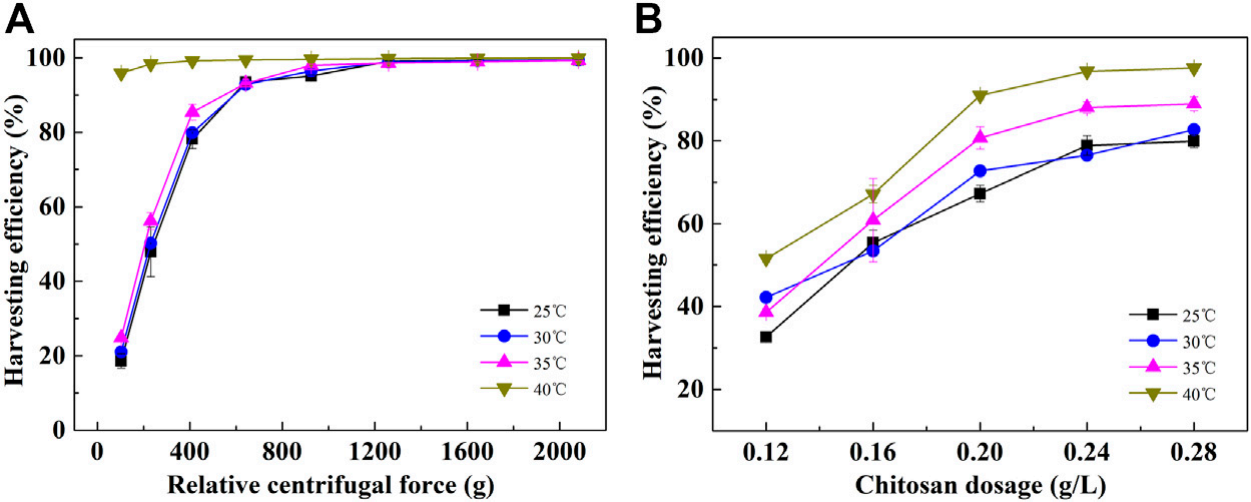
Harvesting efficiency of *C. pyrenoidosa* cultured at different temperatures by centrifugation **(A)** and chitosan flocculation **(B)**. (The time for centrifugation is 2 min, and the settling time after flocculation is 3 min.)

In addition to gravity sedimentation, flocculation and centrifugation are also widely used harvesting methods. As shown in Figure 3, likely, the harvesting efficiency of the cells cultured at 40 °C by flocculation and centrifugation was also significantly higher than that of cells cultured at lower temperatures. It reached 95.87% at the centrifugation speed of 1,000 rpm (103 × *g*). At this condition, the harvesting efficiency of the cells cultured at lower temperatures was only about 20%. To obtain a harvesting efficiency higher than 95%, the centrifugation speed should be more than 3,000 rpm (925 × *g*) for cells cultured at temperatures lower than 40°C (Figure 3A). For flocculation, at a chitosan dosage of 0.20 and 0.24 g/L, the harvesting efficiency of cells cultured at 40°C reached 90.76% and 96.78%, respectively, which was significantly higher than that of cells cultured at low culture temperatures. In addition, the cells cultured at 35°C showed a better harvesting efficiency than the ones cultured at 25 and 30°C. At a chitosan dosage of 0.28 g/L, the harvesting efficiency of cells cultured at 35°C reached 88.93%, while it was only 79.92% at 25°C and 82.71% at 30°C (Figure 3B). Summarily, the energy consumption for centrifugation and flocculant consumption for flocculation were substantially reduced for the harvesting of cells cultured at 40°C. Therefore, cells cultured at high temperatures can effectively reduce the cost of harvesting.

### 3.4 Possible mechanism of high culture temperature promoting cell harvesting

In the process of gravity sedimentation, the settling of the suspended algal cells was derived by gravitational forces on the premise of density difference from the medium. In reality, centrifugation is an extension of gravity sedimentation and only the settling rate was greatly enhanced by the centrifugal force (Roy and Mohanty, 2019). The sedimentation principle can be well explained by Stokes’ law, and the settling rate can be calculated using the following equation (Milledge and Heaven, 2013):

$$Settling\;velocity=\frac{2}{9}g\frac{r^{2}}{\eta }\left(\rho_{s-}\rho_{l}\right),$$ (3)

where η denotes the fluid dynamic viscosity, r denotes the cell radius, *g* denotes the gravitational acceleration, and ρ_s_ and ρ_l_ denote the solid and liquid densities, respectively.

**FIGURE 4:**
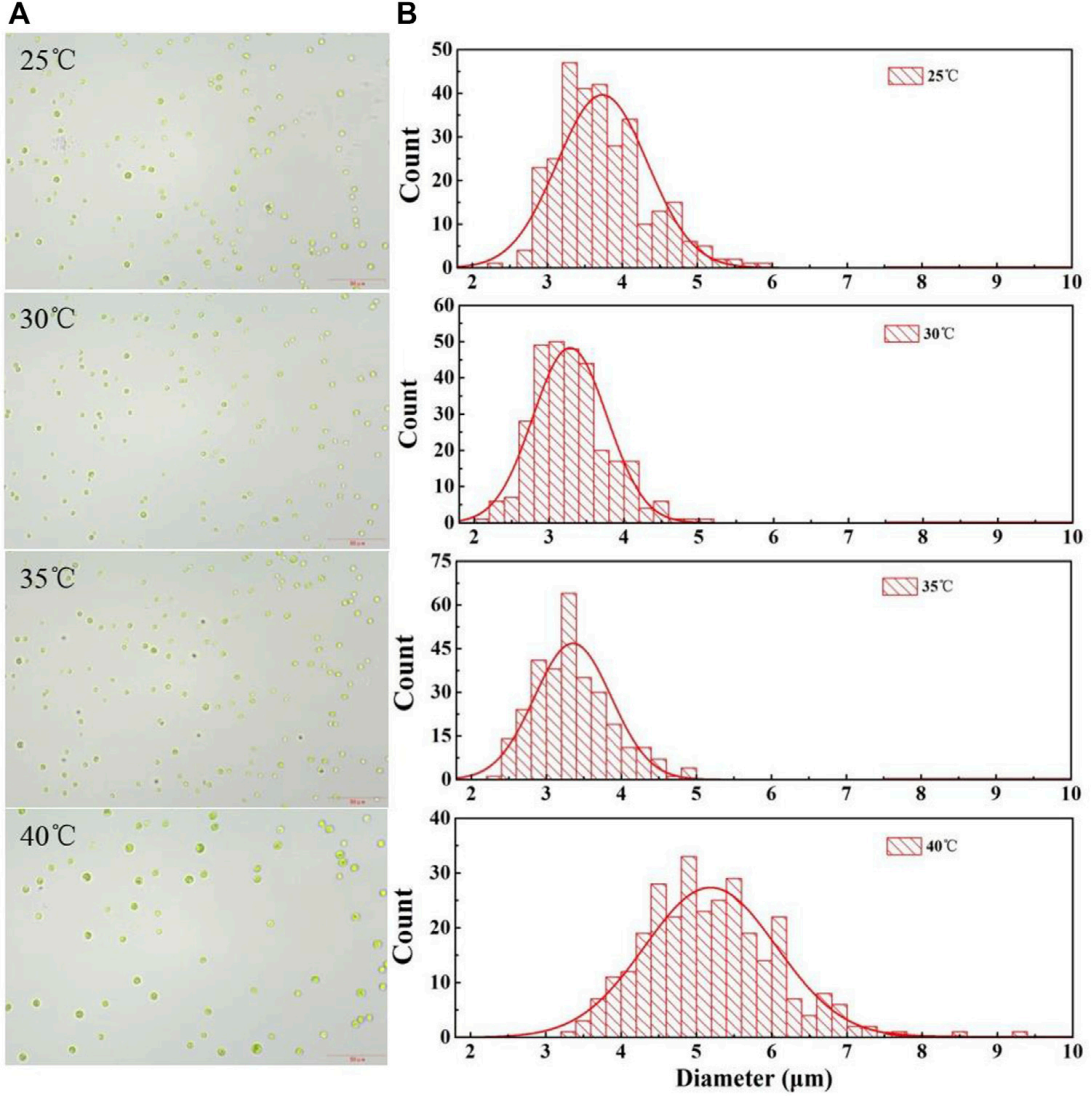
Micrographs **(A)** and cell size distribution **(B)** of *C. pyrenoidosa* after being cultured at different temperatures for 5 days.

The harvesting efficiency of gravity sedimentation and centrifugation depends on the microalgae cell size and density difference between the microalgae biomass and the medium. As shown in Figure 4, there were significant differences in the average cell size at different temperatures after it had been cultured for 5 days. The average diameter of the cells cultured at 25°C reached 3.73 ± 0.60 μm, which is slightly larger than that of cells grown at 30°C (*p* < 0.05), with an average cell diameter of 3.28 ± 0.50 μm. The average size of the cells cultured at 35°C had no significant difference from that of cells cultured at 30°C (*p* > 0.05). However, the average diameter of cells cultured at 40°C was greatly larger than that of cells grown at lower temperatures, reaching 5.19 ± 0.87 μm (Table 2). This result indicated that the cell size was increased under the high culture temperature (Figure 4), which in turn promotes cell harvesting by gravity sedimentation and centrifugation. The enlargement of cell size at higher temperatures was also observed by Barten et al. (2022). They found that the cell volume of *Picochlorum* sp. (BPE23) increased by 46% after an adaptive laboratory evolution at a high temperature for 390 days. However, Yu et al. (2014) detected that the response of cell volume to temperature was species specific. Not all microalgae showed a positive correlation between cell size and temperature. It is noteworthy that most of the research related to the effects of temperature on cell size were conducted in autotrophic modes. The effect of temperature on the heterotrophically cultured algal cells was rarely reported. Therefore, more microalgae should be investigated at different temperatures to confirm whether the positive correlation between cell size and temperature is universal at heterotrophic modes.

**TABLE 2:** Cell average size, pH, and zeta potential of cells cultured at different temperatures at the stationary phase.

Temperature (°C)	Cell diameter (μm)	pH	Zeta potential (mV)
25	3.73 ± 0.60 b	7.60 ± 0.06 c	−23.13 ± 1.23 d
30	3.28 ± 0.50 c	8.32 ± 0.01 a	−21.47 ± 0.49 c
35	3.35 ± 0.51 c	8.33 ± 0.06 a	−18.50 ± 0.92 b
40	5.19 ± 0.87 a	7.94 ± 0.01 b	−7.50 ± 0.29 a

Values in a column with different letters are significantly different according to one-way analysis of variance (ANOVA) (*p* < 0.05).

According to Eq. 3, the density difference between the microalgae biomass and the medium was another key factor for cell settling in the gravity sedimentation and centrifugation processes. Being a biological entity, the most of the composition in algal cells is water. In addition, they can adjust their water content in order to float in the medium freely. As a result, they do not have an exact density (Huang et al., 1999). The cell density is directly influenced by the cell composition as the density of proteins, lipids, and carbohydrates varies greatly. Their average densities are 1,300, 860, and 1,500 kg·m^−3^, respectively (Reynolds, 1984). According to these values, the density of the cells cultured at different temperatures has no significant difference. These indicate that the cell density has little effect on the harvesting efficiency.

The cell size also has a significant influence on flocculation efficiency. Chatsungnoen and Chisti (2016) found that to achieve an equal harvesting efficiency, microalgal species with a larger size required a lower dosage of flocculant than did those with smaller cells. In addition, Vandamme et al. (2010) reported that the dosage of a polymeric flocculant for the effective flocculation of *Scenedesmus* sp. was significantly lower than the dose required for *Parachlorella* sp, as the size of *Scenedesmus* sp. is greatly larger than the later species. This is mainly due to the fact that the cells of a smaller size have higher specific surface areas, thus requiring a higher flocculant dose per unit of biomass weight (Schenk et al., 2008). In this study, the cell size of *C. pyrenoidosa* cultured at 40°C was greatly larger than that of cells grown at the lower temperatures (Table 2). Therefore, less flocculant was required for efficient flocculation.

Due to the ionized functional groups on the cell surface, microalgal cells are generally negatively charged (Ndikubwimana et al., 2015). The stability of a cell suspension is greatly influenced by the cell surface charge. In addition, the surface charge also significantly affects the dosage of an ionic flocculant required to produce effective flocculation (Chatsungnoen and Chisti, 2016). As shown in Table 2, the zeta potential of cells cultured at different temperatures varies greatly. Similar to previous studies, the zeta potential of cells in all cultures was negative. However, its absolute value of cells cultured at 25°C was significantly higher than for others and was continuously decreasing with increasing culture temperature. The zeta potential of cells cultured at 40°C was only −7.5 mV, while its absolute value at other temperatures was more than 18 mV. It has been confirmed that the higher the absolute value of the zeta potential (either positive or negative), the more stable the suspension will remain as individual particles that are strongly repulsing each other (Branyikova et al., 2018). As the absolute value of the zeta potential was quite low at 40°C, harvesting the cells was easier, and the dosage of chitosan, a cationic flocculant, required for effective flocculation was also greatly reduced when compared to that for cells cultured at lower temperatures.

Except for the harvesting methods discussed in this study, flotation, various kinds of filtration, and magnetic separation are also widely used in microalgae harvesting. The harvesting efficiency of these methods is also closely related to cell size, cell density, and the zeta potential (Roy and Mohanty, 2019). The increase in cell size caused by high culture temperatures might also improve the harvesting efficiency of these methods.

### 3.5 Potential advantages

#### 3.5.1 Enhancing cell growth rate

Following the logic of the Arrhenius law, the biological reaction rate and cell growth rate were improved with increasing temperatures within a certain range. In this study, the highest specific growth rate was achieved in cultures at 35 and 40°C. The culture period can be shortened at a higher temperature. This is conducive in improving the utilization of a bioreactor and reducing production costs.

#### 3.5.2 Reducing cost of cell harvesting

Cell harvesting efficiency is closely related to the characteristics of algal cells. It was found that the cell size was significantly increased and the cell surface charge was greatly decreased with the culture temperature increasing to 40°C, which is favorable for cell harvesting by a series of commonly used methods, which include gravity sedimentation, centrifugation, and flocculation. Therefore, the cost of cell harvesting was significantly reduced in these harvesting processes.

#### 3.5.3 Potential application in cultivation and harvesting of other microalgal species

As far as we know, this is the first report that heterotrophically cultured *C. pyrenoidosa* at high temperatures promotes cell harvesting. Currently, the effect of temperature on the heterotrophically cultured algal cells has been rarely reported. The promotion of cell harvesting induced by high culture temperatures might be universal in other microalgal species. This strategy might be widely used for the harvesting of heterotrophically cultured microalgae.

## 4 Conclusion

*Chlorella pyrenoidosa* is a heat-stable microalgae which is capable of growing rapidly at 40°C in the heterotrophic mode. The cells cultured at 40°C were much easier to harvest than those cultured at lower temperatures by gravity sedimentation, centrifugation, and flocculation. The promotion of cell harvesting was mainly attributed to the increased cell size and decreased absolute value of the zeta potential induced by the high culture temperature. This study explored a new approach to simplify heterotrophic cultivation and harvesting of microalgae, which effectively reduces microalgae production costs.

## Data Availability

The raw data supporting the conclusion of this article will be made available by the authors, without undue reservation.
